# Dementia in African American Dyads and Black Immigrant Families: Lessons From IMPACT-AD

**DOI:** 10.1093/geroni/igaf122.1508

**Published:** 2025-12-31

**Authors:** Kalisha Bonds Johnson, Manka Nkimbeng, Joshua Grill, Rema Raman, Kenneth Hepburn, Wizdom Powell, Carol Whitlatch, Karen Lyons

**Affiliations:** Emory University, Atlanta, Georgia, United States; University of Minnesota School of Public Health, Minneapolis, Minnesota, United States; University of California, Irvine, Irvine, California, United States; University of Southern California, Los Angeles, California, United States; Emory University, Atlanta, Georgia, United States; University of Connecticut School of Medicine, Farmington, Connecticut, United States; Benjamin Rose Institute on Aging, Cleveland, Ohio, United States; Boston College, Chestnut Hill, Massachusetts, United States

## Abstract

The goal of IMPACT-AD is to “train the next generation of ADRD clinical trialists, to ensure that investments in discovery yield outcomes for patients and families.” This program offers a weeklong summer workshop with curated sessions. In this presentation, scholars (Summer 2021 and 2022) will discuss the program’s impact on their work and career. Lessons learned include: 1) study design for the development of a clinical trial for dementia dyads (described below) and 2) identifying analytic approaches for behavioral interventions with dementia care partners. Specifically, we will offer outcome data to highlight how IMPACT-AD facilitated the successful implementation of phase one of a mixed-methods intervention to understand the decision self-efficacy (DSE) of dementia care dyads. Data from 39 African American parent-adult daughter dementia dyads was analyzed using multilevel modeling. On average, parents’ DSE was significantly lower than their adult daughters’ DSE. Parents experienced significantly better DSE when they perceived a more positive family decision-making process, reported a positive interaction within the dyadic relationship with their daughter, and had daughters who reported an “intense motivation to succeed.” Adult daughters experienced significantly better DSE when they reported better general family functioning and had greater confidence in their parent’s patient-provider relationship. However, daughters experienced significantly worse DSE when their parents perceived a more positive family decision-making process. Not all scholars’ projects are in the data analysis phase. Cumulatively, scholars gained skills from participation in IMPACT-AD and its annual scientific retreats that they continue to implement in current and planned clinical trial projects.

